# Dissecting HOCl Action in Chronic Wound Biofilms: Proteomic Insights From a Host‐Relevant Model of *Pseudomonas aeruginosa*

**DOI:** 10.1002/mbo3.70181

**Published:** 2025-11-27

**Authors:** Lori I. Robins, Philip Gafken, Chenwei Lin, Lisa Jones, Sarah E. Hooper

**Affiliations:** ^1^ Department of Physical Sciences University of Washington Bothell Washington USA; ^2^ Fred Hutchinson Cancer Research Center Seattle Washington USA; ^3^ Microbiology & Infection Research Group, Cardiff School of Sport and Health Sciences Cardiff Metropolitan University Cardiff UK

**Keywords:** antimicrobial, biofilm, HOCl, proteomics, quorum sensing, virulence

## Abstract

*Pseudomonas aeruginosa* is found in 48%–52% of chronic wound biofilms, where its resistance to antimicrobials and host immunity presents a major clinical challenge. Although hypochlorous acid (HOCl) is known to be an effective antimicrobial, its mechanism of action remains unclear because standard experimental conditions often produce a mixture of HOCl and hypochlorite (OCl⁻), making it difficult to isolate the effects of HOCl. Here, we use proteomic profiling to investigate the effects of a pure, stable HOCl gel on *P. aeruginosa* biofilms in a physiologically relevant chronic wound model. We applied HOCl gel (5.7 mM, pH 6) to mature *P. aeruginosa* biofilms established in a wound‐mimicking flow model. Proteins were analyzed using tandem mass tag (TMT)‐based quantitative proteomics, identifying 1,878 proteins. HOCl treatment significantly reduced biofilm viability and altered the abundance of 330 proteins. We observed substantial depletion of proteins involved in biosynthesis, virulence, antibiotic resistance, and biofilm formation, alongside enrichment of stress response proteins. These findings indicate a shift toward survival phenotypes and weakened pathogenicity. Our data reveal that HOCl disrupts multiple pathways essential for *P. aeruginosa* survival and virulence. Crucially, our experimental design eliminates confounding factors that can lead to unintentional testing of mixed HOCl and OCl⁻ species, allowing us to assess the specific effects of HOCl. These findings call for a re‐evaluation of HOCl research methodologies and reiterate the importance of realistic infection models in antimicrobial testing.

## Graphical abstract

Impact statement:

We tested a gel containing a pure, stable form of hypochlorous acid (HOCl) against *P. aeruginosa*. To ensure our results reflect the true effects of HOCl we used a host‐relevant biofilm model. The HOCl gel disrupted bacterial metabolism, impacted proteins associated with virulence, and changed the abundance antibiotic resistance proteins. These findings suggest the gel could improve healing in chronic wound infections and reduce antibiotic use.

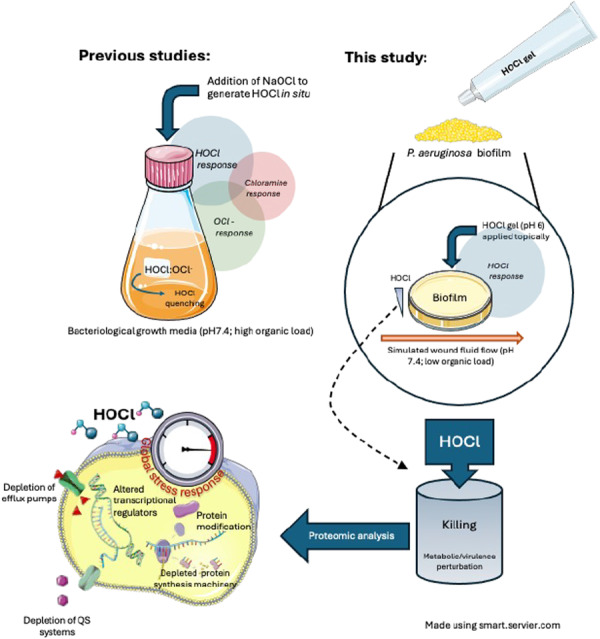

## 1 Introduction

The therapeutic potential of hypochlorous acid (HOCl) in wound care has been recognized since World War I (Beattie et al. 1917). In 1968 the myeloperoxidase‐halide‐hydrogen peroxide antibacterial system was described, and 20 years later, HOCl was confirmed as a key component (Klebanoff 1968; Weiss et al. 1982; Test and Weiss 1986). However, the clinical adoption of HOCl has been hindered by its instability, particularly in early formulations that required on‐site preparation and degraded rapidly (Eryilmaz and Palabiyik 2013). Formulation advances have enabled the production of stable HOCl solutions with extended shelf lives, and additional formulations including gels have renewed interest in its therapeutic potential (Gold et al. 2020; Hughson et al. 2016; Menta et al. 2024; Herruzo et al. 2023).

Early mechanistic studies described how HOCl readily disrupted bacterial membranes, oxidized thiols, and impaired protein synthesis (Knox et al. 1948; Camper and McFeters 1979; Sips and Hamers 1981). Subsequent research revealed that HOCl selectively interacted with cytoplasmic nucleotides and respiratory components, leading to iron loss, oxidative protein damage, and cell death (Albrich et al. 1981; Rosen and Klebanoff 1985, 1982). These downstream effects are thought to be mediated by long‐lasting oxidants (Test and Weiss 1986; Knoke et al. 2024; Winterbourn et al. 2006a). To date, it is hypothesized that bacteriostatic activity, such as halted DNA and protein synthesis, precede bactericidal action (McKenna and Davies 1988). This has been substantiated by global‐expression analyses that link HOCl to redox‐sensitive regulation of biofilm formation and confirm its role in oxidative stress responses (Perkins et al. 2021; El Hajj et al. 2022).

While numerous studies have aimed to describe the mechanism behind the antimicrobial activity of HOCl, our understanding has been limited by the reported use of unstable formulations, pH variability, and diverse experimental platforms that do not mimic real‐world application. Here, we use tandem mass tag (TMT)‐based proteomic analysis to investigate the effects of a stable HOCl gel formulation on *P. aeruginosa* biofilms within a chronic wound model.

Our results provide new mechanistic insights supporting the use of HOCl as a topical treatment for chronic wounds. Importantly, we propose that prior studies may have assessed the antimicrobial effects of mixed solutions of HOCl and OCl⁻ due to variable pH, formulation stability, or test conditions. By contrast, our work isolates and examines the activity of stable HOCl at pH6, under physiologically relevant conditions. Moreover, by using a chronic wound biofilm model that more closely mimics the host environment, we provide a more clinically meaningful understanding of the mode of action of HOCl.

## 2 Materials and Methods

### 2.1 Reagents

For the iodometric titrations, reagents were purchased from Hach (dissolved oxygen 3 powder pillows, potassium iodide powder pillows, sodium thiosulfate digital titrator cartridge (0.113 N), and starch indicator solution). HOCl gel was purchased from Briotech Inc. USA. For the biofilm model, all reagents and media were purchased from Sigma Aldrich.

### 2.2 Bacterial Strains and Culture Conditions

*Pseudomonas aeruginosa* ATCC 9027 was cultured in simulated wound fluid (SWF; 2.34 mM CaCl₂·2H₂O, 3.75 mM KCl, 9.9 mM NaCl, pH 7.4; Sigma Aldrich, UK) containing heat‐inactivated fetal bovine serum (FBS; 3% v/v; Pan‐Biotech, UK) and equilibrated to 1 × 10⁸ CFU/mL. Biofilms were established in agarose‐collagen matrices to mimic the chronic wound environment (Khalid et al. 2023).

### 2.3 Biofilm Flow System

Biofilms were cultured in a custom flow system maintained at 33°C with a flow rate of 0.322 mL/min (Khalid et al. 2023; Duckworth et al. 2018). After 5 h, 0.15 g of HOCl gel or saline gel was applied to the biofilm surface. After 24 h, biofilms were harvested, washed with PBS to remove loosely adherent organisms, quenched with sodium thiosulfate, and homogenized in Laemmli lysis buffer for proteomic analysis. Replicate biofilms were enumerated using the Miles Misra method.

### 2.4 HOCl Gel Concentration Determination

Gel samples (1 mL) were added to 49 mL of 1% NaCl solution in a 50 mL conical vial and vortexed. The active chlorine in the samples was determined by iodometric titrations using sodium thiosulfate (0.113 N) and HACH reagent kits for total (active and free) chlorine (Hach Company, Loveland, CO) following HACH method 8,209.

### 2.5 Pseudomonas Aeruginosa Biofilm TMT Proteomics

**Sample Preparation:** *P. aeruginosa* biofilm samples were divided into two groups; namely, HOCl treated and HOCl untreated samples. The samples were prepared for multiplexed quantitative proteomics analysis following the streamlined TMT (Tandem Mass Tag) protocol of Navarrete‐Navarrete‐Perea et al. (2018) with the HOCl treated samples labeled with TMT 126, 127, and 128 reagents and the untreated samples labeled with TMT 129, 130, and 131 reagents. One change was incorporated into the streamlined TMT protocol where after enzymatic digestion the samples were filtered with a 0.2 µm cellulose acetate spin filter (Agilent) and further washed with 200 µL of 0.1% trifluoro acetic acid. The samples were desalted using Oasis cartridges, taken to dryness, and resuspended in 70 µL of 100 mM HEPES pH 8.5. The streamlined method was then continued by labeling with TMT reagent.

**Mass Spectrometry:** Mass spectrometry data collection was carried out with a ThermoScientific Easy nanoLC 1200 HPLC connected in‐line with an Orbitrap Eclipse with FAIMS (Field Asymmetric Ion Mobility Spectrometry) mass spectrometer. Data dependent acquisition was performed with SPS‐RTS (Synchronous Precursor Scanning with Real Time Search).

**Data Analysis:** Collected data were processed with the ThermoScientific Proteome Discoverer v2.5 software package. The data were searched against a *P. aeruginosa* ATCC 9027 database that included common contaminants (cRAPome). Searches were performed with settings for the proteolytic enzyme trypsin. Maximum missed cleavages were set to 2. The precursor ion tolerance was set to 10 ppm and the fragment ion tolerance was set to 0.5 Da. Dynamic peptide modifications were set for oxidation on methionine (+ 15.995 Da) and modifications on the protein N‐terminus, consisting of acetylation (+ 42.011 Da), Met‐loss (−131.040 Da), and Met‐loss+Acetyl (−89.030 Da). Static modifications were set for TMT on any N‐terminus (+ 229.163 Da), TMT on lysine (+ 229.163 Da), and carbamidomethyl on cysteine (+ 57.021 Da). Sequest HT was used for protein database searching and Percolator was used for peptide validation.

Raw quantitative results were transformed to log2 scale and normalized to the median value across samples. Missing data were imputed with half of the global minimum value. P‐values for two sample (control vs. HOCl) comparisons were calculated by *t*‐test and all plots were created by ggplot2 in R.

### 2.6 Functional Clustering Analysis

To reduce bias in functional designation a Web of Science search using the terms “*Pseudomonas aeruginosa* proteomics” was done. This returned 846 peer‐reviewed publications. These were filtered using the exclusion criteria indicated in Supporting Information S1: Table S1. Criteria in the remaining 22 manuscripts informed the functional groups and designations in this study.

## 3 Results

### 3.1 Bacterial Viability

**Figure 1:**
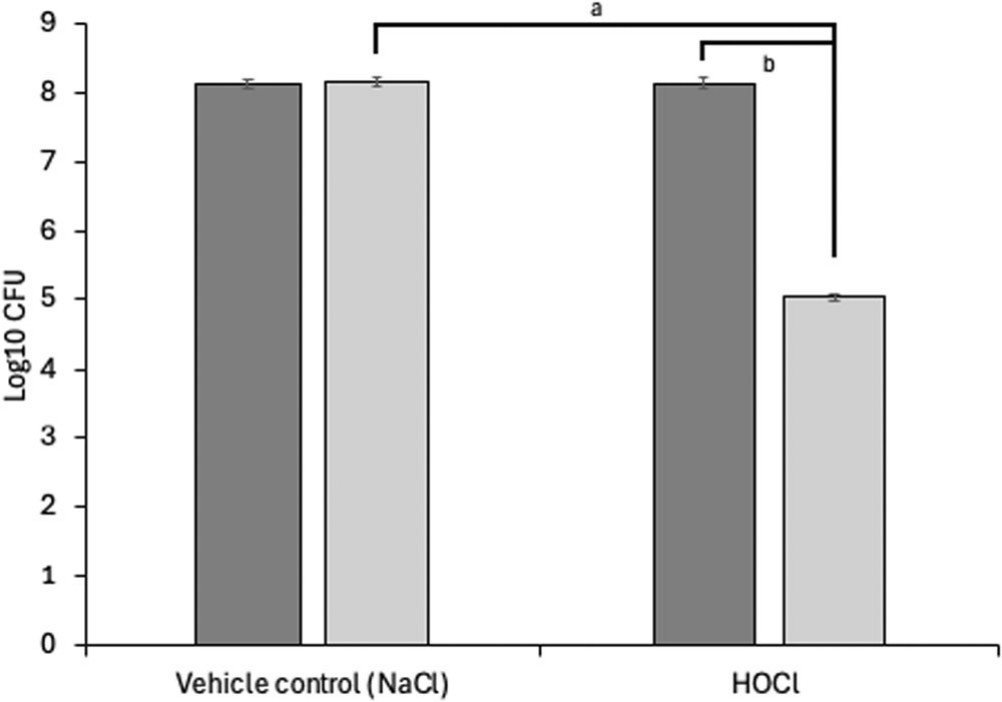
Viable *P. aeruginosa* after treatment with the HOCl gel. HOCl reduced the numbers of viable bacteria by approximately 3‐log 24 h posttreatment. The vehicle control was NaCl gel; HOCl refers to the HOCl gel (5.7 mM). Dark gray: T = 0; light gray: T = 24. SEM are shown and comprise N = 3 and *n* = 4. The following relevant comparisons indicate a statistically significant reduction (*p* < 0.05) in log_10_ for (a) HOCl at T = 24 compared to the vehicle control at T = 24 and (b) HOCl at T = 24 compared to HOCl at T = 0. Analysis used 2‐way ANOVA and Tukey's *post‐hoc* test.

Before proteomic analysis, the viability of *P. aeruginosa* in our biofilm model was tested in the presence of 0.15 g of 5.7 mM topically applied HOCl gel. As expected, HOCl treatment reduced biofilm viability by approximately three log‐fold, with treated biofilms showing an average viable count of 1.12 × 10⁵ CFU/mL compared to 1.46 × 10⁸ CFU/mL in controls (Figure 1).

### 3.2 Proteomic Analysis

**Figure 2:**
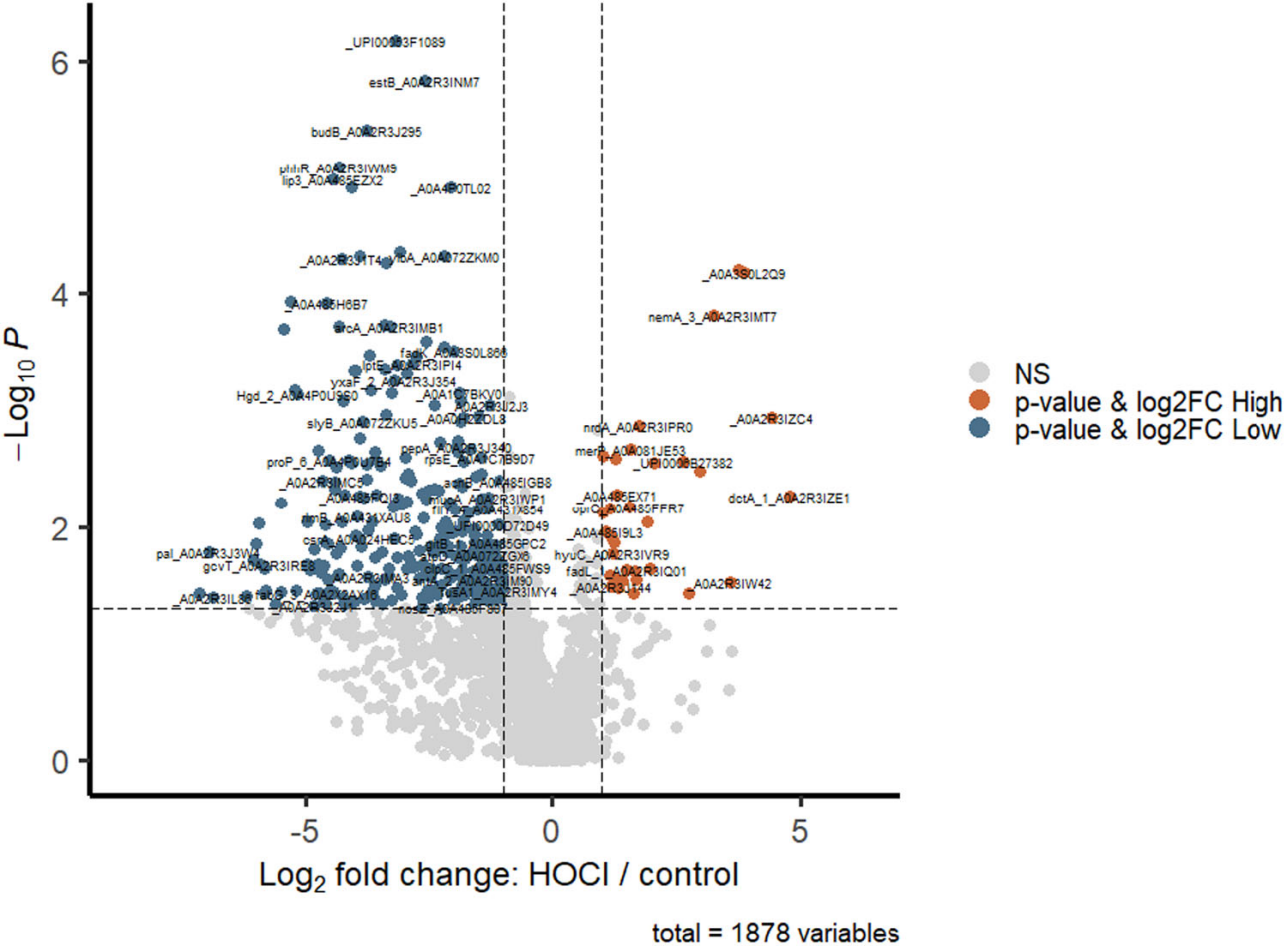
Volcano plot representing differentially expressed proteins between HOCl treated and control samples [*p* < 0.05 and > 2‐fold difference].

Analysis using TMT for multiplexing of *P. aeruginosa* biofilms identified significant differences (*p* < 0.05) in protein expression between HOCl (5.7 mM) treated and control biofilms. A total of 1878 proteins were identified and of these, 297 of these proteins had decreased abundance and 33 were elevated in response to HOCl. We used a twofold change cut‐off and *p* < 0.05 threshold for significance to identify key protein changes (Figure 2; Supporting Information S1: Table S2).

### 3.3 Enriched Proteins Predominantly Comprise a Global “Rescue” Response

Proteins with known roles in global bacterial stress responses, cell integrity, detoxification, and DNA repair were differentially identified in response to HOCl (> 2‐fold, *p* < 0.05) (Supporting Information S1: Table S3). Among these proteins, 60% showed enrichment while 40% exhibited depletion. Notably enriched were NemA and MmcQ, known for their roles in HOCl and chlorine stress, alongside three proteins involved in the export of ROS, metal ions, and heme.

### 3.4 Proteins Associated With Normal Cellular Functioning and Antibiotic Responses Are Depleted by HOCl Treatment

Key proteins involved in protein biosynthesis, virulence, biofilm formation including quorum sensing, and motility were significantly depleted (> 2‐fold; *p* < 0.05) (Supporting Information S1: Tables S4 and S5). Of these, 44.2% were identified as ribosomal proteins, 17.3% involved with biofilm processes, 15.3% associated with virulence, and 11.5% with motility and transcription/translation. Collectively, these proteins are vital for normal cell functioning and infection. No proteins associated with any of these categories were significantly enriched after treatment with HOCl.

Analysis of the global data identified 32 depleted proteins associated with antibiotic and antimicrobial responses (Supporting Information S1: Table S6). These proteins have functions that are known targets for six different classes of antibiotics that affect ribosomal subunits, DNA gyrase, peptidoglycan biosynthesis, and LPS biosynthesis and transport (Table 1).

**Table 1:** Antibiotic target proteins depleted after HOCl treatment.

Family of antibiotics	Known targets in bacteria	*P. aeruginosa* proteins altered by exposure to HOCl (proteomic analysis)
Aminoglycosides	Ribosomal 30 s	RpsE, RpsD, RpsF, RpsS, RpsU, RpsL, RpsK, RpsP (small ribosomal subunits)
Quinolones	DNA gyrase and synthesis via topoisomerase II and IV	PmbA (modulator of DNA gyrase: PmbA/TldE family)
B‐lactams	Penicillin binding proteins	CpoB (peptidoglycan biosynthesis during cell division) Penicillin binding protein activator (LpoA homolog)
Polymyxins	Lipid/LPS biosynthesis	LpxC (Lipid A biosynthesis) FabG2 (LPS biosynthesis)
Macrolides	Ribosomal 50 s	RplB, RplX, RplC, RplO, RplS, RplV, RplM, RpmG, RmpE, RmpG, RmpE2, RplU (large ribosomal subunits)
Macrocyclic peptide (MCP)	LPS transport	LptE (LPS assembly lipoprotein)

## 4 Discussion

HOCl is a powerful oxidant produced by activated neutrophils at concentrations ranging between 25 and 50 mM in vivo (Winterbourn et al. 2006b; Summers et al. 2008; Epstein and Weiss 1989). It reacts readily with pathogens via interactions with macromolecules, particularly proteins where it can modify amino acid side chains including cysteine, methionine, lysine, and histidine (Ulfig and Leichert 2021; Anees et al. 2021; Andrés et al. 2022; Sultana et al. 2020; Pattison and Davies 2001). These oxidative modifications have the ability to disrupt protein structure, impair enzymatic function, and compromise essential cellular processes (Hawkins 2020). Previous studies have explored the global impact of HOCl on bacterial cells where it is hypothesized that HOCl both easily penetrates and reacts rapidly with the cell envelope (Knoke et al. 2024; Fukuzaki 2006). It is also possible that the effect is mediated by long‐lasting oxidants (Test and Weiss 1986; Knoke et al. 2024; Winterbourn et al. 2006a). Documented cytoplasmic effects include perturbation of the respiratory chain, protein synthesis, DNA replication, biofilm formation, and virulence. Furthermore, HOCl is thought to inactivate or deplete bacterial oxidative defences, making bacteria more susceptible to damage (Dukan et al. 1999). This study explored the protein‐level changes in *P. aeruginosa* after topical HOCl exposure in a realistic chronic wound biofilm model. These findings not only advance our understanding of the antimicrobial mechanism of HOCl but also challenge longstanding assumptions shaped by experimental designs, that may not clearly differentiate between the effects of HOCl and OCl⁻.

In *P. aeruginosa*, an exogenously applied mixture of HOCl/NaOCl at pH 7.5, is reported to mediate expression changes for numerous genes associated with oxidative stress, membrane integrity and transport, metabolism, growth and DNA repair (Chen et al. 2021; Small et al. 2007; Farrant et al. 2020; Nontaleerak et al. 2020). Differences to metabolic biomarkers (e.g., virulence factors) related to the *P. aeruginosa* quinolone signal (PQS) quorum sensing system have also been detected after treatment with 3.2 mM NaOCl diluted into growth media (Bradley et al. 2024). Our analysis identified 33 significantly (*p* < 0.05; > 2‐fold change) enriched proteins, and 297 significantly (*p* < 0.05; > 2‐fold change) depleted proteins, following treatment with 5.7 mM HOCl as a topical gel at pH 6. Of those proteins with a significant change, only one (FlgM) overlapped with a prior study of *P. aeruginosa* that used transposon mutants exposed to 4.4–5.1 mM HOCl (Farrant et al. 2020). We observed an overlap with four proteins (CynT, FliS, LeuA, SlyB) that were identified in *Escherichia coli* by differential gene expression using 2 mM NaOCl, and with six proteins (NemA, YedY, CsgG, ClpA, Trx family, Nrd family), identified by RNA‐Seq in uropathogenic *E. coli* (UPEC) exposed to 2.25 mM NaOCl at pH 12, suggesting some targets are conserved among Gram‐negative bacteria (Chen et al. 2021; Sultana et al. 2022). Given the essential nature of most of these differentially regulated genes and proteins, a higher degree of conservation among components of the global response to HOCl is expected.

The observed differences and lack of conservation are likely due to the pH of the active chlorine solution, the bacteriological growth media, experimental design, and the analytical techniques. At pH 7.5, both HOCl and NaOCl are present in equal amounts as active chlorine species where HOCl is 80‐100 times more potent than NaOCl (Kiamco et al. 2019). In broth/planktonic culture, much of the HOCl reacts directly with the growth media, with is likely to either quench the active chlorine or generate long lasting oxidants such as chloramines, which are known to be less reactive than HOCl (Peskin and Winterbourn 2001). The subsequent blend of oxidative species arising from this method has the potential to significantly influence the outcomes, particularly in terms of accurately attributing activity and mechanistic insights exclusively to HOCl. Our experimental setup differs, allowing for direct contact between HOCl and the *P. aeruginosa* biofilm while minimizing interactions with the simulated wound fluid in our flow model. The dimensions of the substratum in which biofilms were cultured (5 mm depth, 8 mm diameter) combined with the flow rate (2 ml/h) results in fluid replacement every 7.6 min. It is possible that this allows for the removal of any chloramine byproducts and neutralized HOCl, with the topical gel providing a concentrated, localized dose. Critically, the HOCl gel used in this study was pH 6 with greater than 96% of the active chlorine species present as HOCl (Mehendale et al. 2023).

Our data concurs with current hypotheses for the global stress response of Gram‐negative bacteria to HOCl/NaOCl, showing significant enrichment of chlorine‐specific stress adaptation and detoxification proteins, alongside oxidative stress response proteins. For example, the oxidoreductase NemA, a detoxification protein, repressed by the transcriptional regulator NemR, was enriched under our experimental conditions (Farrant et al. 2020; Gray et al. 2013a, 2013b). Gene expression analysis in *E. coli* agrees with our findings where the expression of *nemA* increased in response to 2.25 mM NaOCl (Sultana et al. 2022). In *E. coli*, NemR, dissociates from DNA following oxidation of Cys116 residue in the presence of HOCl (Gray et al. 2013a, 2013b; Feige 2018). HOCl‐mediated oxidation of cysteine on NemR is likely responsible for the increased abundance of NemA we observe by TMT analysis (Subhadra et al. 2019). An uncharacterized protein that mapped to the MmcQ/YjbR protein family was similarly enriched in our data. MmcQ is a member of the MarR class of redox sensor proteins involved in the resistance to reactive oxygen species, often using oxidation of cysteines for sensing (Grove 2017; Xuan et al. 2024). We hypothesize that the protein identified in our study, is likely involved with *P. aeruginosa* survival in the presence of HOCl by quenching the active chlorine. Eleven other proteins associated with oxidative stress protection and membrane integrity were depleted. Structural analysis suggests that these proteins contain reactive groups (‐SH, ‐NH, ‐NH₂), making them highly susceptible to specific HOCl‐induced damage. Such modifications can initiate bacterial protein degradation pathways, which may account for some of the depletion seen in this study. It is also conceivable that the modified fragments were not detected by our method leading to lower protein abundance. Both possibilities are the result of direct modification of these proteins. Interestingly, we do not observe an enrichment of chaperone proteins, which is reported for *E. coli* and *Vibrio cholerae* in the presence of active chlorine (Winter et al. 2008; Wholey and Jakob 2012).

It is well documented that HOCl can react with membrane components to destabilize the cell envelope (Barrette et al. 1987; Spickett et al. 2000). The Msr system, for example, in Gram‐negative bacteria comprises up to five proteins (MsrA, MsrB, MsrC, MsrP, and BisC) that help repair the cell envelope (Andrieu et al. 2020). MsrP, crucial for cell envelope maintenance, showed significant enrichment in our study. A previous study in *E. coli* showed that another Msr protein (MsrA) repairs HOCl‐induced damage, however, we did not observe significant enrichment of this protein (Rosen et al. 2009). In *P. aeruginosa*, MsrA is constitutively expressed, unlike in *E. coli* where it is regulated by oxidative stress which may explain this observation. However, our previous studies, suggest that exogenous HOCl can overwhelm these functional repair systems, to mediate complete bacterial killing in our biofilm model (Nedelea et al. 2022).

HOCl modification of membrane‐bound proteins can disrupt ATP biosynthesis and respiration (Albrich et al. 1981; Sultana et al. 2020; Barrette et al. 1989; Flurin et al. 2021). Following HOCl treatment, we detected a significant depletion of several cytochrome c peroxidases and oxidoreductases despite their cytoplasmic localization (see Supplemental Information). Their redox‐reactive side chains likely make them highly susceptible to HOCl modification, contributing to impaired respiration and energy production. Oxidation of methionine, the heme ligand, and iron in the presence of HOCl has been observed for cytochrome c (Prütz et al. 2001; Chen et al. 2002). Redox‐sensing regulatory proteins are crucial for the bacterial oxidative stress response. OxyR, a key redox stress regulator in *P. aeruginosa*, has been linked to HOCl stress responses in *E. coli*, *Salmonella spp*., and *Xanthomonas campestris (*Bismuth et al. 2021; Charoenlap et al. 2015; Gundlach and Winter 2014). Previous studies show increased *oxyR* transcription under HOCl and NaOCl exposure, yet our data reveal OxyR protein depletion following HOCl treatment (Dukan and Touati 1996). Depletion in our experimental design is probably due to modifications of amino acids, aggregation or degradation of the protein. OxyR is activated by disulfide bond formation between Cys 199 and Cys 208 upon oxidation and requires methionine and histidine for activity (Pedre et al. 2018; Johnson et al. 2006). All of these residues are susceptible to HOCl oxidation which plausibly leads to a decrease in detection in our experiment, and further investigations to determine if specific modifications have occurred are necessary, to more completely understand the mechanism of action. We saw significant enrichment of DctA, which is involved in c4‐dicarboxylic acid transport, and regulated in part by the ArcAB redox regulon, suggesting a complex interplay between oxidative stress, respiration and energy production for bacteria exposed to HOCl (Loui et al. 2009; Davies et al. 1999).

Our data show that HOCl disrupts key *P. aeruginosa* survival pathways and targets 32 proteins linked to antibiotic resistance (Table 1), including efflux pumps conferring resistance to cephalosporins and aminoglycosides, and AmrR, an anti‐repressor associated with aminoglycoside impermeability. This suggests that HOCl has the potential to enhance antibiotic sensitivity, offering synergy between topical and systemic treatments. However, we also found that MacB, a macrolide exporter responsive to oxidative stress, was significantly enriched, which may protect *P. aeruginosa* from macrolides or require multiple doses of exogenous HOCl as seen in our previous studies (Nedelea et al. 2022). Analyses of early *P. aeruginosa* biofilms exposed to sub‐lethal NaOCl, indicate that HOCl enhances attachment and biofilm formation (Strempel et al. 2017). This is supposed to provide protection against oxidative stress and is thought to be a conserved response to biocide treatment (Charron et al. 2023). Yet in our chronic wound model, the opposite occurs. We observed significant depletion of proteins involved in attachment, quorum sensing, and EPS production, aligning with total viable count data that indicate impaired *P. aeruginosa* biofilms. This conforms to case study observations where topical HOCl reduces infection in chronic ulcers *in vivo (*Herruzo et al. 2023 *)*. Our prior work on five‐species chronic wound biofilms also supports these findings, suggesting that the impact of HOCl on biofilm formation is context‐dependent (Khalid et al. 2023; Nedelea et al. 2022). Beyond its effects on antibiotic resistance and biofilm disruption, HOCl also impacts bacterial virulence. Some studies suggest HOCl activates virulence systems to resist phagosomal killing, but we observed depletion of key virulence factors, including those involved in the flagellar apparatus, type III and type VI secretion systems, and exotoxin A. HOCl treated bacteria likely prioritize survival over pathogenicity, aligning with effective immune clearance that is observed in vivo. By targeting critical bacterial pathways HOCl emerges as a powerful therapeutic for chronic wound care. Direct application of a stable formulation that remains at the wound site, provides a practical solution for managing chronic infections. Experimental topicals using in situ electrochemical generation of HOCl have shown strong efficacy against wound pathogens, reinforcing its potential as an effective treatment (Kiamco et al. 2019; Flurin et al. 2021). Our results with localized HOCl in gel formulation allowed for the direct analysis of the impacts of a single active chlorine species and confirm its use in the treatment of chronic wounds. The simplicity of this formulation makes it well‐suited for widespread adoption, particularly in low‐ and middle‐income countries where the socioeconomic burden of chronic wounds is high.

While this study provides detailed protein‐level insights, our ongoing work on phenotypic validation will complement these proteomic analyses, further strengthening the mechanistic understanding of HOCl‐mediated modulation of virulence and resistance. However, our current findings raise important considerations for future research. First, there is a need to re‐examine previous studies that attributed effects to HOCl without verifying speciation. Second, the divergence between simple biofilm or planktonic models and our more physiologically representative wound model suggests that model choice significantly influences observed antimicrobial effects. In our system, HOCl triggered both direct bacterial killing and potentially deeper sub‐lethal perturbations in metabolism and virulence, which may support host clearance. These nuances are unlikely to be captured in oversimplified models, and we encourage the field to adopt more clinically realistic platforms to guide therapeutic development.

## Author Contributions

**Lori I. Robins:** concept, experimental design, experimental work, design of project, data analysis, writing and reviewing manuscript. **Philip Gafken:** experimental work, data analysis, writing and reviewing manuscript. **Chenwei Lin:** experimental work, data analysis, writing and reviewing manuscript. **Lisa Jones:** experimental work, writing and reviewing manuscript. **Sarah E. Hooper:** concept, experimental design, experimental work, design of project, data analysis, writing and reviewing manuscript.

## Ethics Statement

The authors have nothing to report.

## Conflicts of Interest

The authors declare no conflicts of interest.

## Supporting information


**Table S1:** Identifying journal articles for analysis. **Table S2:** Summary of all proteins with differential expression. > 2‐fold decreased abundance = ‐1; >2‐fold increased abundance = 1. **Table S3:** Proteins with differential abundance associated with stress and survival. **Table S4:** Proteins with differential abundance associated with virulence and infection. **Table S5:** Proteins with differential abundance associated with protein synthesis. **Table S6:** Proteins with differential abundance associated with antibiotic targets and resistance.
