# Supplementary material for: Dissecting HOCl Action in Chronic Wound Biofilms: Proteomic Insights From a Host‐Relevant Model of Pseudomonas aeruginosa
Source: Microbiologyopen. 2025 Nov 27;14(6):e70181. doi: 10.1002/mbo3.70181 (PMC12658613; doi:10.1002/mbo3.70181)
Supplement: Supplementary file 1 — Table S1: Identifying journal articles for analysis. Table S2: Summary of all proteins with differential expression. > 2‐fold decreased abundance = ‐1; >2‐fold increased abundance = 1. Table S3: Proteins with differential abundance associated with stress and survival. Table S4: Proteins with differential abundance associated with virulence and infection. Table S5: Proteins with differential abundance associated with protein synthesis. Table S6: Proteins with differential abundance associated with antibiotic targets and resistance. [file MBO3-14-e70181-s001.docx]

Supplemental Information

[Table S1. Identifying journal articles for analysis. 2](#_Toc192595462)

[Table S2. Summary of all proteins with differential expression. >2-fold decreased abundance = -1; >2-fold increased abundance = 1. 2](#_Toc192595463)

[Table S3. Proteins with differential abundance associated with stress and survival. 16](#_Toc192595464)

[S4. Proteins with differential abundance associated with virulence and infection. 17](#_Toc192595465)

[Table S5. Proteins with differential abundance associated with protein synthesis. 18](#_Toc192595466)

[Table S6. Proteins with differential abundance associated with antibiotic targets and resistance. 19](#_Toc192595467)

### Table S1. Identifying journal articles for analysis.

| Searched/excluded terms | Number of Journal Articles |
| --- | --- |
| *Pseudomonas aeruginosa* proteomics | 846 |
| Pseudomonas aeruginosa AND proteomics | 476 |
| *Pseudomonas aeruginosa* | 304 |
| Secretome, extracellular, vesicles, exoproteome, ECM | 280 |
| Named antibiotics, MDR, AMR, tolerance | 235 |
| Phage OR bacteriophage | 227 |
| Review, mathematical OR predictive model | 220 |
| Environmental *Pseudomonas* species | 189 |
| Co-culture studies OR bacterial interactions | 177 |
| Host-pathogen, pathoadaptation, immune, vaccine | 138 |
| Transcriptome | 139 |
| Gene knockouts, CRISPR, protein overexpression | 125 |
| Protein interaction studies | 123 |
| Suitable analysis OR targeted focus | 22 |

### Table S2. Summary of all proteins with differential expression. >2-fold decreased abundance = -1; >2-fold increased abundance = 1.

| Protein | Differential expression | Function |
| --- | --- | --- |
| acnB_A0A485IGB8 | -1 | aconitate hydratase 2 |
| clpC_1_A0A485FWS9 | -1 | ClpA/B rotease ATP binding subunit |
| atpD_A0A072ZGX6 | -1 | ATP synthase beta chain |
| fusA1_A0A2R3IMY4 | -1 | elongation factor G |
| _UPI0000D72D49 | -1 | *Pseudomonas aeruginosa* ABC transporter substrate-binding protein |
| ggt1_A0A2R3IWC9 | -1 | gamma-glutamyltranspeptidase |
| _UPI00053D3745 | -1 | *Pseudomonas aeruginosa* partial elongation factor Tu |
| _A0A2R3J2J3 | -1 | nitrate reductase (quinone) |
| _A0A2R3ISW0 | -1 | fumarate hydratase class I |
| arcA_A0A2R3IMB1 | -1 | arginine deaminase |
| _UPI0006E59183 | -1 | alpha-2-macroglobulin homolog |
| rho_A0A069Q0W1 | -1 | transcription termination factor |
| algC_A0A485GG03 | -1 | phosphomannomutase |
| sthA_A0A2R3IWS8 | -1 | soluble pyridine nucleotide transhydrogenase |
| fliY_4_A0A431X854 | -1 | L-cysteine transporter of ABC system FLiY |
| nucA_A0A485HRV4 | -1 | endonuclease |
| _UPI0006B26EB0 | -1 | ribonuclease E |
| pepA_A0A2R3J340 | -1 | leucine aminopeptidase |
| nosZ_A0A485F807 | -1 | nitrous oxide reductase precursor |
| rplC_A0A072ZBZ2 | -1 | 50S ribosomeal protein L3 |
| atpF_A0A2R3IM05 | -1 | ATP synthase subunit (E. coli) |
| osmY_2_A0A071L3C8 | -1 | osmoprotectant import permease protein OsmY (*E. coli*) |
| lpxC_A0A2R3J0I2 | -1 | UDP-3-O-acyl-N-acetylglucosamine deacetylase |
| dsbA_A0A2R3IVE0 | -1 | [thiol:disulfide interchange protein DsbA](https://www.pseudomonas.com/feature/show?id=113862) |
| pilB_A0A3S0IX76 | -1 | type 4 fimbrial biogenesis protein |
| opuAC_4_A0A485F5N5 | -1 | glycine-betaine-binding protein |
| _A0A485GYZ9 | -1 | periplasmic/secreted protein |
| _A0A485GA16 | -1 | dimethylglycine catabolism protein DgcA |
| gltB_1_A0A485GPC2 | -1 | [glutamate synthase large chain precursor](https://www.pseudomonas.com/feature/show?id=112942) |
| rpsE_A0A1C7B9D7 | -1 | 30S ribosomal protein S2 |
| yebE_A0A2R3IVY9 | -1 | transmembrane protein |
| mucD_A1YSK5 | -1 | [serine protease MucD precursor](https://www.pseudomonas.com/feature/show?id=104282) |
| infC_A0A431X4F6 | -1 | translation initiation factor IF-3 |
| mmsA_A0A2R3INC7 | -1 | [methylmalonate-semialdehyde dehydrogenase](https://www.pseudomonas.com/feature/show?id=109959) |
| _A0A2R3J281 | -1 | DUF2138 domain-containing protein |
| rhlE_1_A0A485FVX0 | -1 | ATP-dependent RNA helicase RhlE |
| rplB_A0A1C7BR18 | -1 | 50S ribosomal protein L2 |
| antA_2_A0A2R3IM90 | -1 | anthranilate dioxygenase large subunit |
| accC1_A0A2R3IXT2 | -1 | biotin carboxylase |
| hemL_A0A2R3IM23 | -1 | glutamate-1-semialdehyde 2,1-aminomutase |
| tagQ_A0A2R3J271 | -1 | type VI secretion system-associated lipoprotein TagQ |
| oprF_2_A0A2R3ITG1 | -1 | major porin and structural outer membrane porin OprF precursor |
| _A0A2R3INS9 | -1 | lipoprotein |
| sdhB_A0A2R3J177 | -1 | [succinate dehydrogenase (B subunit)](https://www.pseudomonas.com/feature/show?id=105938) |
| mqo_2_A0A3S0L7B2 | -1 | probable malate:quinone oxidoreductase 2 |
| dppA_4_A0A2R3J200 | -1 | di/tripeptide-binding protein 4 |
| _A0A2R3IX49 | -1 | LysM domain protein |
| _UPI0006B267BA | -1 | adenylosuccinate lyase |
| _UPI0006B26D57 | -1 | penicillin-binding protein activator |
| rnr_A0A485GQ02 | -1 | [exoribonuclease RNase R](https://www.pseudomonas.com/feature/show?id=112740) |
| ppk_A0A485GDI2 | -1 | polyphosphate kinase |
| rplX_A0A1C7B7C3 | -1 | 50S ribosomal protein L24 |
| groES_A0A071KZF6 | -1 | GroES protein |
| hupA_A0A0H2ZJ52 | -1 | DNA-binding protein HU-alpha |
| rpsD_A0A2R3IU40 | -1 | 30S ribosomal protein S4 |
| rpe_A0A2R3J348 | -1 | [ribulose-phosphate 3-epimerase](https://www.pseudomonas.com/feature/show?id=103953) |
| _A0A2R3J4E1 | -1 | curli production assembly/transport component CsgG |
| hppD_A0A2R3IXD3 | -1 | 4-hydroxyphenylpyruvate dioxygenase |
| fabG_3_A0A2X2AX16 | -1 | 3-oxoacyl-[acyl-carrier-protein] reductase |
| ccpA_A0A485GVK5 | -1 | cytochrome c551 peroxidase |
| _UPI0006B26135 | -1 | AAA family ATPase |
| icmF1_A0A485G3Z8 | -1 | transmembrane protein |
| _UPI0006B2A34A | -1 | sarcosine oxidase subunit alpha |
| _UPI0006E659E0 | -1 | serine/threonine protein kinase PpkA, partial |
| rplO_A0A1C7BKS0 | -1 | 50S ribosomal protein L15 |
| mlaF_A0A072ZCA4 | -1 | peptidylprolyl isomerase |
| himA_A0A024HFA3 | -1 | [integration host factor, alpha subunit](https://www.pseudomonas.com/feature/show?id=108264) |
| ndk_A0A069Q6F8 | -1 | nucleoside diphosphate kinase |
| rpsF_A0A069Q263 | -1 | 30S ribosomal protein S6 |
| ybaK_3_A0A2R3IRL3 | -1 | conserved hypothetical protein |
| dppF_A0A2R3J581 | -1 | [dipeptide ABC transporter ATP-binding protein DppF](https://www.pseudomonas.com/feature/show?id=111850) |
| _A6V1W0 | -1 | putative ATP-dependent RNA helicase |
| uvrB_A0A2R3IUR1 | -1 | [excinuclease ABC subunit B](https://www.pseudomonas.com/feature/show?id=109088) |
| cpoB_A0A2R3J3K1 | -1 | cell division coordinator CpoB |
| _A0A431WZY1 | -1 | ATPase |
| _UPI00053ECF6F | -1 | alcohol dehydrogenase |
| edd_A0A2R3ITM4 | -1 | [phosphogluconate dehydratase](https://www.pseudomonas.com/feature/show?id=109201) |
| wzz_A0A485F494 | -1 | O-antigen chain length regulator |
| citB_A0A485H7T9 | -1 | aconitate hydratase |
| rplS_A0A1C7BMI3 | -1 | 50S ribosomal protein L19 |
| _A0A0H2ZDL8 | -1 | putative periplasmic inhibitor/zinc-resistance associated protein |
| oxyR_A0A2R3ISW2 | -1 | OxyR protein |
| _A0A2R3IX92 | -1 | copper-translocating P-type ATPase |
| _A0A2R3J2G7 | -1 | DUF4197 domain-containing protein |
| metZ_A0A2R3IU37 | -1 | o-succinylhomoserine sulfhydrylase |
| _A0A485H5M5 | -1 | YceI-like domain-containing protein |
| _A0A2R3IMR7 | -1 | methionine biosynthesis MetW family protein |
| yfkM_1_A0A2R3IU52 | -1 | PfpI family protein |
| accA1_2_A0A485IIH9 | -1 | acetyl-CoA carboxylase, biotin carboxylase |
| narH_A0A2R3J3V1 | -1 | respiratory nitrate reductase beta chain |
| pheT_A0A485ITR9 | -1 | phenylalanyl-tRNA synthetase, beta subunit |
| _A0A2R3IWF4 | -1 | dipeptidase |
| _A0A485FQI3 | -1 | UDP-N-acetylmuramoyl-tripeptide--D-alanyl-D-alanine ligase |
| murF_A0A2R3J0T0 | -1 | [UDP-N-acetylmuramoylalanyl-D-glutamyl-2, 6-diaminopimelate--D-alanyl-D-alanyl ligase](https://www.pseudomonas.com/feature/show?id=111670) |
| rplV_A0A010SEM6 | -1 | 50S ribosomal protein L22 |
| hfq_A0A0V8SYY9 | -1 | Hfq protein |
| rpsS_A0A0V8T064 | -1 | 30S ribosomal protein S19 |
| rpsU_A0A024HBP4 | -1 | 30S ribosomal protein S19 |
| _A0A2R3IVC1 | -1 | membrane integrity-associated transporter subunit PqiC |
| rluC_A0A2R3IRY2 | -1 | pseudouridine synthase |
| adiA_A0A2R3IN26 | -1 | [ribosomal large subunit pseudouridine synthase C](https://www.pseudomonas.com/feature/show?id=108746) |
| dppA_3_A0A2R3IQ88 | -1 | ABC transporter binding protein |
| tssB_A0A3S0J109 | -1 | type VI secretion protein |
| moaC_A0A2R3IZR0 | -1 | molybdopterin biosynthetic protein C |
| rpsI_A0A2R3J3W0 | -1 | 30S ribosomal protein S9 |
| rplM_A0A2R3INL4 | -1 | 50S ribosomal protein L13 |
| _A0A2R3J204 | -1 | Rho termination factor, N-terminal domain protein |
| _A0A2R3ISY3 | -1 | thioredoxin peroxidase |
| _A0A2R3J1F5 | -1 | nucleoprotein/polynucleotide-associated enzyme |
| elbB_A0A2R3IYE3 | -1 | glyoxalase |
| _UPI00053DCF62 | -1 | delta-aminolevulinic acid dehydratase |
| lptE_A0A2R3IPI4 | -1 | LPS-assembly lipoprotein LptE |
| tolB_A0A485HDD7 | -1 | TolB protein |
| _A0A485GAA0 | -1 | secreted protein |
| _A0A2R3IPB8 | -1 | putative outer membrane protein |
| _UPI0006B279E4 | -1 | xanthine dehydrogenase molybdopterin binding subunit |
| ppiA_A0A485F510 | -1 | peptidyl-prolyl cis-trans isomerase A |
| nadE_A0A485GM26 | -1 | [NH3-dependent NAD synthetase](https://www.pseudomonas.com/feature/show?id=112706) |
| infA_A0A010SV25 | -1 | initiation factor |
| ribB_A0A0H2ZEN7 | -1 | [GTP cyclohydrolase II / 3,4-dihydroxy-2-butanone 4-phosphate synthase](https://www.pseudomonas.com/feature/show?id=110928) |
| secB_A0A072ZJB5 | -1 | secretion protein secB |
| gbcB_A0A072ZFE6 | -1 | GbcB protein glycine-betaine catabolism; tolerance to hyperosmotic stress |
| ftsA_A0A071L3J7 | -1 | cell division protein ftsA |
| rpsK_A0A024HAW1 | -1 | 30S ribosomal protein S11 |
| rpsP_A0A1S1BVD4 | -1 | 30S ribosomal protein S11 |
| _A0A2R3IS46 | -1 | DctP family TRAP transporter solute-binding subunit |
| _A0A2R3J2B6 | -1 | HDOD domain protein |
| _A0A431XFU9 | -1 | dienelactone hydrolase family protein |
| pyrF_A0A2R3J0R4 | -1 | orotidine 5'-phosphate decarboxylase |
| puo_A0A2R3IQC5 | -1 | flavin monoamine oxidase family protein |
| panC_A0A3S0J1Y5 | -1 | pantoate-beta-alanine ligase |
| trpC_A0A2R3J352 | -1 | [indole-3-glycerol-phosphate synthase](https://www.pseudomonas.com/feature/show?id=104041) |
| purK_A0A2R3J1Q9 | -1 | [phosphoribosylaminoimidazole carboxylase](https://www.pseudomonas.com/feature/show?id=113734) |
| _UPI00053D062E | -1 | RhiA protein |
| eda_1_A0A4P0UD79 | -1 | 2-dehydro-3-deoxy-phosphogluconate aldolase |
| _UPI00053EDCE2 | -1 | putrescine binding protein |
| purM_A0A485HH21 | -1 | [phosphoribosylaminoimidazole synthetase](https://www.pseudomonas.com/feature/show?id=104652) |
| _UPI0006B27717 | -1 | imidazolonepropionase |
| capD_A0A4P0UDQ9 | -1 | involved in antibiotic resistance |
| csrA_A0A024HEC5 | -1 | RsmA |
| rpmG_A0A0H2ZJ31 | -1 | [50S ribosomal protein L33](https://www.pseudomonas.com/feature/show?id=113504) |
| _A0A2R3IP05 | -1 | bacterial regulatory, TetR family protein |
| gshB_A0A2R3ISE1 | -1 | [glutathione synthetase](https://www.pseudomonas.com/feature/show?id=103551) |
| mucD_2_A0A2R3J0Y7 | -1 | [serine protease MucD precursor](https://www.pseudomonas.com/feature/show?id=104282) |
| _A0A2R3IY67 | -1 | alpha/beta hydrolase |
| fadK_A0A3S0L866 | -1 | fatty acid beta-oxidation |
| mucA_A0A2R3IWP1 | -1 | [anti-sigma factor MucA](https://www.pseudomonas.com/feature/show?id=104276) |
| rebM_A0A2R3IZL5 | -1 | biosynthetic pathway of 7-Halo-tryptophan, pyrrolnitrin and rebeccamycin; RebM, methyltransferase |
| ubiG_A0A2R3ISB3 | -1 | [3-demethylubiquinone-9 3-methyltransferase](https://www.pseudomonas.com/feature/show?id=109155) |
| _A0A2R3IQK5 | -1 | transmembrane protein |
| ftsX_A0A2R3IUY6 | -1 | cell division protein FtsX |
| rlmI_2_A0A4P0TJ95 | -1 | ribosomal large subunit – methyltransferase activity |
| fklB_1_A0A4P0TGN2 | -1 | [peptidyl-prolyl cis-trans isomerase FklB](https://www.pseudomonas.com/feature/show?id=111990) |
| _A0A485ISL8 | -1 | SMC domain-containing protein |
| blaPDC_A0A173G7Y2 | -1 | oxyimino-cephalosporin resistance |
| psiF_A0A485GPY6 | -1 | [conserved hypothetical protein](https://www.pseudomonas.com/feature/show?id=112614) |
| _UPI0006E66022 | -1 | acetyl-coA-C-acyltransferase |
| fabG_6_A0A431X989 | -1 | [3-oxoacyl-[acyl-carrier-protein] reductase](https://www.pseudomonas.com/feature/show?id=108730) |
| nqrD_A0A2R3INE4 | -1 | Na+-translocating NADH:uniquinone oxidoreductase subunit Nqr4 |
| rpmE_A0A2R3J325 | -1 | [50S ribosomal protein L31](https://www.pseudomonas.com/feature/show?id=1674354) |
| trpG_A0A2R3ISP2 | -1 | anthranilate synthase component II |
| _A0A485IUQ9 | -1 | bacteriophage protein |
| _A0A424XAL4 | -1 | ACT domain-containing protein |
| _A0A2R3J1B4 | -1 | lactonase, 7-bladed beta-propeller family protein |
| ydjA_A0A485F5B8 | -1 | nitroreductase family protein |
| _A0A2R3IMB2 | -1 | DUF2135 domain-containing protein |
| _UPI0006B270D3 | -1 | TIR domain-containing protein |
| _A0A485FYJ8 | -1 | uncharacterized protein conserved in bacteria |
| spoOJ_A6VF40 | -1 | chromosome partitioning protein Spo0J |
| osmE_A0A2R3IMZ7 | -1 | [DNA-binding transcriptional activator OsmE](https://www.pseudomonas.com/feature/show?id=1673978) |
| _A0A2R3IM51 | -1 | aldehyde dehydrogenase |
| _A0A2R3IMA3 | -1 | pyrroloquinoline quinone (Coenzyme PQQ) biosynthesis protein C |
| moaB_1_A0A2R3J0G5 | -1 | molybdenum cofactor biosynthesis protein B |
| _UPI0005B9100A | -1 | gluconate 2-dehydrogenase, membrane bound, cytochrome c |
| _A0A2R3IWR8 | -1 | cytochrome C; electron transfer activity; heme binding; iron binding |
| xthA_2_A0A2R3IQW5 | -1 | exodeoxyribonuclease III |
| pkn1_2_A0A485IRN7 | -1 | type II secretory system/virulence |
| _A0A2R3IR82 | -1 | DUF2384 domain-containing protein |
| _UPI0006E4F668 | -1 | type VI secretion system associated FHA domain protein TagH |
| gcvT_A0A2R3IRE8 | -1 | [glycine cleavage system aminomethyltransferase T](https://www.pseudomonas.com/feature/show?id=1674700) |
| hemX_A0A431X4H1 | -1 | heme biosynthesis operon protein HemX |
| _A0A485IJ42 | -1 | putative cysteine proteases |
| arsC1_A0A485HFC6 | -1 | putative arsenate reductase |
| nuoF_A0A485IU35 | -1 | [NADH dehydrogenase I subunit F](https://www.pseudomonas.com/feature/show?id=1667921) |
| _A0A3S0JJP0 | -1 | Aldehyde dehydrogenase |
| ppiC_1_A0A2R3J3T6 | -1 | Peptidyl-prolyl cis-trans isomerase |
| ccp_A0A485F0S7 | -1 | cytochrome *c* peroxidase |
| rlmB_A0A431XAU8 | -1 | 23S rRNA (guanosine-2'-O-)-methyltransferase RlmB |
| _A0A2R3ILQ9 | -1 | Acyl-CoA dehydrogenase, N-terminal domain protein |
| fabB_A0A2R3IS94 | -1 | 3-oxoacyl-ACP synthase |
| pal_A0A2R3J3W4 | -1 | peptidoglycan associated outer membrane protein |
| _A0A485FEN3 | -1 | periplasmic transport protein |
| armR_A0A1G7UIG4 | -1 | ArmR (beta-lactam resistance) |
| hpt_A0A071L0F2 | -1 | hypoxanthine-guanine phosphoribosyltransferase |
| gcvT_A0A2R3IWW0 | -1 | [glycine cleavage system aminomethyltransferase T](https://www.pseudomonas.com/feature/show?id=1674700) |
| hemY_A0A2R3ITA2 | -1 | heme metabolic process |
| ubiB_A0A2R3IZ14 | -1 | 2-polyprenylphenol 6-hydroxylase |
| leuC_A0A2R3ISV9 | -1 | isopropylmalate isomerase large subunit |
| proP_6_A0A4P0U7B4 | -1 | proline/betaine transporter |
| _A0A2R3IMC5 | -1 | heme-oxygenase-associated N-terminal helices domain-containing protein |
| uvrC_A0A431XAE5 | -1 | excinuclease ABC subunit C |
| _A0A4P0TNQ4 | -1 | amino acid ABC transporter substrate-binding protein |
| _A0A485G607 | -1 | beta alanine--pyruvate transaminase |
| _UPI0006B280E5 | -1 | amidase |
| actP_A0A2R3J2B2 | -1 | acetate permease |
| _A0A485IW63 | -1 | putative secretion system protein |
| ilvH_A0A0H2ZHP5 | -1 | [acetolactate synthase 3 regulatory subunit](https://www.pseudomonas.com/feature/show?id=1673600) |
| algU_A0A0H2Z5X2 | -1 | RNA polymerase sigma factor AlgU |
| ruvA_A0A072ZZE7 | -1 | Holliday junction DNA helicase RuvA |
| aroE_A0A431XJC8 | -1 | [shikimate 5-dehydrogenase](https://www.pseudomonas.com/feature/show?id=1662844) |
| _A0A2R3J1R6 | -1 | lipoprotein |
| pyrB_A0A2R3IYY9 | -1 | [aspartate carbamoyltransferase catalytic subunit](https://www.pseudomonas.com/feature/show?id=1663796) |
| _A0A2R3J2J1 | -1 | cupin |
| nirF_A0A2R3IWJ5 | -1 | [heme d1 biosynthesis protein NirF](https://www.pseudomonas.com/feature/show?id=1664026) |
| _A0A2R3IL86 | -1 | lipoprotein |
| _A0A2R3ITB9 | -1 | pilin assembly protein |
| mcpA_A0A424XH87 | -1 | methyl-accepting chemotaxis protein McpA |
| fleN_A0A140SDQ3 | -1 | flagellar synthesis regulator FleN |
| yaeQ_A0A431XCY3 | -1 | uncharacterized protein; suppresses RfaH defect |
| panB_1_A0A4P0TFG9 | -1 | [3-methyl-2-oxobutanoate hydroxymethyltransferase](https://www.pseudomonas.com/feature/show?id=1673674) |
| _A0A2R3IQ73 | -1 | L-serine dehydratase |
| _A0A2R3J0P7 | -1 | Dyp-type peroxidase family protein |
| pyrC_2_A0A485G0D7 | -1 | dihydroorotase |
| _UPI00053EACEA | -1 | hypothetical protein |
| _A0A4P0TFI7 | -1 | helix-turn-helix domain-containing protein |
| _A0A2R3INU8 | -1 | acyl-CoA dehydrogenase |
| _UPI0006E51008 | -1 | wall associated protein |
| aruF_A0A072ZN39 | -1 | arginine/ornithine succinyltransferase AI subunit |
| rpmB_A0A072ZIQ9 | -1 | [50S ribosomal protein L28](https://www.pseudomonas.com/feature/show?id=1674964) |
| trmD_A0A072ZPS1 | -1 | tRNA (guanine-N(1)-)-methyltransferase |
| rbfA_A0A2R3J2C1 | -1 | ribosome-binding factor A |
| pcaQ_A0A2R3J2Y3 | -1 | [transcriptional regulator PcaQ](https://www.pseudomonas.com/feature/show?id=1663252) |
| _A0A2R3J4S8 | -1 | DotU family type VI secretion system protein (OmpA/MotB family protein) |
| gsiB_A0A2R3J337 | -1 | glucose starvation-inducible protein B |
| flhF_A0A2R3IXY8 | -1 | flagellar biosynthesis regulator FlhF |
| _A0A0C7D4G9 | -1 | UPF0235 protein CAZ10_35455 |
| phrB_A0A2R3J2C3 | -1 | deoxyribodipyrimidine photolyase |
| _A0A2R3IWH8 | -1 | type II secretion system protein M |
| _A0A431XG26 | -1 | CAP domain-containing protein |
| _A0A485F1W8 | -1 | protein BatD |
| ku_A0A2R3IZR5 | -1 | non-homologous end joining protein Ku |
| proV_1_A0A2R3IXL6 | -1 | putative ABC transporter |
| _UPI0006B2788A | -1 | ATP-dependent protease |
| _UPI0006B29C1A | -1 | hemerythrin-like domain containing protein |
| ybiC_A0A485HRR8 | -1 | (S)-2-haloacid dehalogenase |
| kipI_2_A0A485ILE6 | -1 | sporulation inhibitor KipI |
| Hgd_2_A0A4P0U9S0 | -1 | 3-hydroxyisobutyrate dehydrogenase; valine catabolic process |
| _A0A2R3J3V9 | -1 | tRNA threonylcarbamoyl adenosine modification protein, Sua5/YciO/YrdC/YwlC family; double stranded RNA binding |
| _A0A2R3J1T4 | -1 | ABC transporter substrate-binding protein; Mce family protein (methyl accepting chemotaxis protein) |
| hisS_A0A2R3J1K1 | -1 | [histidyl-tRNA synthetase](https://www.pseudomonas.com/feature/show?id=1665415) |
| _A0A485IHH8 | -1 | fumarylacetoacetase |
| lip3_A0A485EZX2 | -1 | Lip3 protein; hydrolase alpha/beta fold family |
| yjbJ_A0A0H2ZGT7 | -1 | CsbD family protein |
| rpsN_A0A1C7BR27 | -1 | small ribosomal subunit protein |
| fliS_A0A2R3IM20 | -1 | bacterial type flagellum assembly (export chaperone) |
| _A0A2R3J0J2 | -1 | glutathione S-transferase |
| pyrR_A0A2R3IVJ1 | -1 | bifunctional pyrimidine regulatory protein PyrR/uracil phosphoribosyltransferase |
| yecD_A0A2R3IU44 | -1 | isochorismatase family protein YecD |
| budB_A0A2R3J295 | -1 | acetolactate synthase |
| _UPI00068C9728 | -1 | *Pseudomonas aeruginosa* protein-L-isoaspartate O-methyltransferase |
| _A0A485F596 | -1 | ABC-type transport system protein |
| potF_4_A0A485G1W7 | -1 | putrescine binding periplasmic protein |
| pmbA_A0A485GYR1 | -1 | putative modulator of DNA gyrase |
| _UPI0006B28568 | -1 | probable 3-mercaptopyruvate sulfurtransferase |
| _A0A485H6B7 | -1 | putrescine binding periplasmic protein |
| rhdA_A0A485GMX2 | -1 | [thiosulfate sulfurtransferase](https://www.pseudomonas.com/feature/show?id=1674152) |
| _A0A4P0U934 | -1 | zinc-type alcohol dehydrogenase-like protein |
| slyB_A0A072ZKU5 | -1 | outer membrane lipoprotein SlyB |
| ylbA_A0A072ZKM0 | -1 | allantoin catabolism protein |
| _A0A1C7BKV0 | -1 | transcriptional regulator |
| echA8_4_A0A1G7UKK9 | -1 | probable enoyl-CoA hydratase |
| phhR_A0A2R3IWM9 | -1 | transcriptional regulator PhhR |
| leuA_A0A431XCS9 | -1 | 2-isopropylmalate synthase |
| ptxS_A0A2R3J1N9 | -1 | transcriptional regulator PtxS |
| flhB_A0A2R3IXP7 | -1 | [flagellar biosynthesis protein FlhB](https://www.pseudomonas.com/feature/show?id=1670544) |
| _A0A2R3IZD4 | -1 | glucose starvation-inducible protein B |
| rpmE2_A0A2R3IY21 | -1 | 50S ribosomal protein L31 |
| _A0A1Y0GHW2 | -1 | DUF2845 domain-containing protein |
| clpP_A0A2R3IWI0 | -1 | ATP-dependent Clp protease proteolytic subunit |
| _A0A2R3IYN1 | -1 | DUF4398 domain-containing protein |
| _A0A2R3IWB7 | -1 | DUF469 domain-containing protein |
| _UPI00053B8DFF | -1 | biotin carboxyl carrier protein of acetyl-CoA carboxylase |
| _UPI00053D389A | -1 | LTTR HTH domain containing transcriptional regulator |
| _A0A2R3IVK3 | -1 | iron-containing alcohol dehydrogenase family protein |
| _A0A485I4W1 | -1 | oxidoreductase |
| _A0A4P0TL02 | -1 | putative chemotaxis protein |
| yxaF_2_A0A2R3J354 | -1 | uncharacterized HTH-ype transcriptional regulator; homologous to lmrA |
| _UPI00053F0826 | -1 | DUF2066 domain-containing protein |
| _UPI00053F1089 | -1 | cyanophycin synthase |
| actIII_A0A2R3IV78 | -1 | ketoacyl reductase |
| estB_A0A2R3INM7 | -1 | carboxylesterase 2 |
| _UPI0005BDE43F | -1 | phage tail protein |
| _A0A485EVW6 | -1 | uncharacterized protein |
| _UPI0006B2710F | -1 | lipase secretion chaperone |
| glpQ_1_A0A485G0F0 | -1 | glycerophosphoryl diester phosphodiesterase |
| _A0A485IVZ8 | -1 | lipoprotein |
| cynT_1_A0A485G2J0 | -1 | carbonate dehydratase |
| _UPI0006B27382 | 1 | filamentous haemagglutinin |
| nrdA_A0A2R3IPR0 | 1 | catalytic component of class Ia ribonucleotide reductase |
| fadL_1_A0A2R3IQ01 | 1 | probable outer membrane protein precursor |
| _A0A2R3J144 | 1 | DUF1329 domain-containing protein (lipoprotein localization family) |
| rplU_A0A2R3IZT6 | 1 | 50S ribosomal protein L21 |
| flgD_A0A2R3IUS3 | 1 | [flagellar basal-body rod modification protein FlgD](https://www.pseudomonas.com/feature/show?id=104924) |
| _A0A485I9L3 | 1 | uncharacterized conserved protein |
| _A0A485IGB9 | 1 | membrane protein |
| yajC_A0A0H2ZF92 | 1 | conserved hypothestical protein |
| flgM_A0A2R3IU20 | 1 | FlgM protein |
| bkdA1_A0A2R3IP50 | 1 | 2-oxoisovalerate dehydrogenase (alpha subunit) |
| oprP_1_A0A2R3ILJ3 | 1 | [phosphate-specific outer membrane porin OprP precursor](https://www.pseudomonas.com/feature/show?id=109375) |
| _A0A4P0TF87 | 1 | chromosome segregation ATPase |
| oprC_A0A485FFR7 | 1 | putative copper transport outer membrane porin OprC precursor |
| rsfS_A0A2R3IXF5 | 1 | ribosomal silencing factor |
| hyuC_A0A2R3IVR9 | 1 | N-carbamoyl-beta-alanine amidohydrolase |
| yedY_1_A0A485GTD8 | 1 | msrP; protein-methinine-sulfoxide reductase subunit |
| merP_A0A081JE53 | 1 | mercuric transport protein periplasmic component |
| trxB_2_A0A4P0TMH2 | 1 | thioredoxin reductase (*E. coli*) |
| _UPI0005BB8B22 | 1 | DUF262 domain-containing protein |
| ynfD_1_A0A2R3J1L1 | 1 | putative outer membrane protein |
| ccmA_2_A0A485I6R3 | 1 | [heme exporter protein CcmA](https://www.pseudomonas.com/feature/show?id=105718) |
| _A0A485EX71 | 1 | ABC transporter permease |
| _UPI0006B27A79 | 1 | D-serine hydratase |
| nadC_A0A485GY39 | 1 | nicotinate-nucleotide pyrophosphorylase |
| gbpA_1_A0A2R3IWC4 | 1 | chitin binding protein (CbpD) |
| kgtP_2_A0A2R3J2N4 | 1 | [dicarboxylic acid transporter PcaT](https://www.pseudomonas.com/feature/show?id=103193) |
| _A0A2R3IW42 | 1 | uncharacterized protein conserved in bacteria |
| dctA_1_A0A2R3IZE1 | 1 | [C4-dicarboxylate transport protein](https://www.pseudomonas.com/feature/show?id=105134) |
| _A0A2R3IZC4 | 1 | cupin domain-containing protein; (S)-ureidoglycine aminohydrolase cupin domain-containing protein |
| _A0A3S0L2Q9 | 1 | 4-hydroxy-3-methylbut-2-enyl diphosphate reductase |
| nemA_3_A0A2R3IMT7 | 1 | N-ethymaleimide reductase (*E. coli*) |
| macB_3_A0A485IRL0 | 1 | macrolide export ATP-binding/permease protein |

### Table S3. Proteins with differential abundance associated with stress and survival.

| Protein ID | STRESS AND SURVIVAL |
| --- | --- |
| ***> 2-fold increase*** | ***Global stress response*** |
| nemA_3_A0A2R3IMT7 | adaptive response to HOCl exposure |
| yjbJ_A0A0H2ZGT7 | CsbD; stress response protein |
| yjbR_A0A2R31W42 | contributes to reactive chlorine species stress response (MmcQ) |
| kgtP_2_A0A2R3J2N4 | PcaT; osmotic shock response |
| oprC_A0A485FFR7 | ion transport and stress response |
| nadC_A0A485GY39 | oxidative stress response |
| ***> 2-fold reduction*** |  |
| oxyR_A0A2R3ISW2 | activates the expression of a regulon of H_2_O_2_-inducible genes |
| gbcB_A0A072ZFE6 | tolerance to hyperosmotic stress |
| _A0A2R3IZD4 | stress-induced bacterial acidophilic repeat motif family protein |
| ***>2-fold increase*** | ***Cell integrity*** |
| fadL_1_A0A2R3IQ01 | essential for synthesis of membrane lipids |
| yajC_A0A0H2ZF92 | maintenance of cell envelope |
| ***> 2-fold reduction*** |  |
| _A0A2R3IVC1 | membrane integrity associated transporter |
| oprF_2_A0A2R3ITG1 | major porin and structural outer membrane porin OprF precursor |
| tolB_A0A485HDD7 | cell envelope integrity |
| wzz_A0A485F494 | OM assembly (HOCl disrupts assembly of OM) |
| ***>2-fold increase*** | ***Detoxification*** |
| macB_3_A0A485IRL0 | multidrug efflux pump (macrolides);  detoxifies extracellular reactive oxygen species by export of linearised siderophores |
| yedY_1_A0A485GTD8 | MsrP; protein-methionine-sulfoxide reductase subunit |
| ccmA_2_A0A485I6R3 | heme ABC exporter |
| merP_A0A081JE53 | mercuric ion/metal ion transport protein |
| trxB_2_A0A4P0TMH2 | thioredoxin reductase |
| _A0A485I4W1 | putative FAD-linked oxidoreductase |
| ***>2-fold increase*** | ***DNA repair*** |
| nrdA_A0A2R3IPR0 | DNA synthesis and repair in response to oxidative stress |
| ***>2-fold decrease*** |  |
| hupA_A0A0H2ZJ52 | stabilizes DNA under extreme environmental stress |
| uvrB_A0A2R3IUR1 | catalyzes recognition and processing of DNA lesions |
| uvrC_A0A431XAE5 | catalyzes recognition and processing of DNA lesions |

### S4. Proteins with differential abundance associated with virulence and infection.

| Protein ID | VIRULENCE & INFECTION |
| --- | --- |
| ***> 2-fold decrease*** | ***Attachment, EPS biosynthesis, and quorum sensing*** |
| A0A2R3ITB9 | pilin assembly protein |
| A0A2R3J4E1 | curli production/transport |
| algU_A0A0H2Z5X2 | RNA polymerase sigma factor (virulence, motility, EPS production) |
| csrA_A0A024HEC5 | RsmA; quorum-sensing and regulation of single species biofilm |
| hfq_A0A0V8SYY9 | global virulence regulator; quorum sensing |
| mucA_A0A2R3IWP1 | anti-sigma factor MucA |
| mucD_2_A0A2R3J0Y7 | MucD pre-cursor |
| oprF_2_A0A2R3ITG1 | major porin - transport biofilm matrix proteins |
| pilB_A0A3S0IX76 | type IV fimbrial biogenesis |
| ***> 2-fold decrease*** | ***Motility*** |
| fleN_A0A140SDQ3 | flagella synthesis regulator |
| flhB_A0A2R3IXP7 | flagellar biosynthesis protein |
| flgM_A0A2R3IU20 | negative regulator of flagellin |
| flhF_A0A2R3IXY8 | flagella synthesis regulator |
| fliY_A0A431X854 | flagellar motor switch |
| flgD_A0A2R3IUS3 | flagellar hook formation |
| ***> 2-fold decrease*** | ***Virulence*** |
| A0A2R3J4S8 | DotU family type VI secretion system protein (OmpA/MotB family protein) |
| hfq_A0A0V8SYY9 | involved globally in regulating virulence and quorum sensing |
| icmF1_A0A485G3Z8 | transmembrane protein (type VI secretion system) |
| pkn1_A0A485IRN7 | type III secretory system |
| ptxS_A0A2R3J1N9 | exotoxin A production |
| tagH_UPI0006E4F668 | type VI secretion system-associated FHA domain protein |
| tagQ_A0A2R3J271 | type VI secretion system-associated lipoprotein |
| UPI006E59183 | alpha-2-macroglobulin homologue |

### Table S5. Proteins with differential abundance associated with protein synthesis.

| Protein ID | PROTEIN BIOSYNTHESIS |
| --- | --- |
| ***> 2-fold decrease*** | ***Ribosomal Proteins*** |
| rpsK_A0A024HAW1 | small ribosomal subunit protein uS11 |
| rpsP_A0A1S1BVD4 | small ribosomal subunit protein bS16 |
| rpsS_A0A0V8T064 | 30S ribosomal protein S19 |
| rpsU_A0A024HBP4 | 30S ribosomal protein S19 |
| rpsE_A0A1C7B9D7 | 30S ribosomal protein S2 |
| rpsD_A0A2R3IU40 | 30S ribosomal protein S4 |
| rpsF_A0A069Q263 | 30S ribosomal protein S6 |
| rpsI_A0A2R3J3W0 | 30S ribosomal protein S9 |
| rplM_A0A2R3INL4 | 50S ribosomal protein L13 |
| rplO_A0A1C7BKS0 | 50S ribosomal protein L15 |
| rplS_A0A1C7BMI3 | 50S ribosomal protein L19 |
| rplB_A0A1C7BR18 | 50S ribosomal protein L2 |
| rplU_A0A2R3IZT6 | 50S ribosomal protein L21 |
| rplV_A0A010SEM6 | 50S ribosomal protein L22 |
| rplX_A0A1C7B7C3 | 50S ribosomal protein L24 |
| rpmB_A0A072ZIQ9 | [50S ribosomal protein L28](https://www.pseudomonas.com/feature/show?id=1674964) |
| rpmE_A0A2R3J325 | [50S ribosomal protein L31](https://www.pseudomonas.com/feature/show?id=1674354) |
| rpmE2_A0A2R3IY21 | 50S ribosomal protein L31 |
| rpmG_A0A0H2ZJ31 | [50S ribosomal protein L33](https://www.pseudomonas.com/feature/show?id=113504) |
| rlmI_2_A0A4P0TJ95 | ribosomal RNA large subunit methyltransferase I |
| adiA_A0A2R3IN26 | [ribosomal large subunit pseudouridine synthase C](https://www.pseudomonas.com/feature/show?id=108746) |
| rsfS_A0A2R3IXF5 | ribosomal silencing factor |
| rpsN_A0A1C7BR27 | small ribosomal subunit protein |
| ***> 2-fold decrease*** | ***Elongation Factors*** |
| fusA1_A0A2R3IMY4 | Elongation factor G |
| _UPI00053D3745 | *Pseudomonas aeruginosa* partial elongation factor Tu |
| ***> 2-fold decrease*** | ***Translation Initiation Factors*** |
| infC_A0A431X4F6 | translation initiation factor IF-3 |
| infA_A0A010SV25 | initiation factor |
| ***> 2-fold decrease*** | ***Aminoacyl-tRNA Synthetases*** |
| hisS_A0A2R3J1K1 | histidyl-tRNA synthetase |
| pheT_A0A485ITR9 | phenylalanyl-tRNA synthetase, beta subunit |

### Table S6. Proteins with differential abundance associated with antibiotic targets and resistance.

| Protein ID | ANTIBIOTIC TARGETS & RESISTANCE |
| --- | --- |
| ***> 2-fold decrease*** | ***Targets shared with aminoglycosides*** |
| rpsD_A0A2R3IU40 | small ribosomal subunits (Ribosomal 30S) |
| rpsE_A0A1C7B9D7 | small ribosomal subunits (Ribosomal 30S) |
| rpsF_A0A069Q263 | small ribosomal subunits (Ribosomal 30S) |
| rpsK_A0A024HAW1 | small ribosomal subunits (Ribosomal 30S) |
| rpsI_A0A2R3J3W0 | small ribosomal subunits (Ribosomal 30S) |
| rpsN_A0A1C7BR27 | small ribosomal subunits (Ribosomal 30S) |
| rpsP_A0A1S1BVD4 | small ribosomal subunits (Ribosomal 30S) |
| rpsS_A0A0V8T064 | small ribosomal subunits (Ribosomal 30S) |
| rpsU_A0A024HBP4 | small ribosomal subunits (Ribosomal 30S) |
| ***> 2-fold decrease*** | ***Targets shared with beta-lactams*** |
| cpoB_A0A2R3J3K1 | peptidoglycan biosynthesis |
| UPI0006B26D57 | penicillin binding protein activator (LpoA) |
| ***> 2-fold decrease*** | ***Targets shared with macrocyclic peptides (MCPs)*** |
| lptE_A0A2R3IPI4 | LPS assembly lipoprotein (LPS transport) |
| ***> 2-fold decrease*** | ***Targets shared with macrolides*** |
| rplB_A0A1C7BR18 | large ribosomal subunits (Ribosomal 50S) |
| rplC_A0A072ZBZ2 | large ribosomal subunits (Ribosomal 50S) |
| rplM_A0A2R3INL4 | large ribosomal subunits (Ribosomal 50S) |
| rplO_A0A1C7BKS0 | large ribosomal subunits (Ribosomal 50S) |
| rplS_A0A1C7BMI3 | large ribosomal subunits (Ribosomal 50S) |
| rplU_A0A2R3IZT6 | large ribosomal subunits (Ribosomal 50S) |
| rplV_A0A010SEM6 | large ribosomal subunits (Ribosomal 50S) |
| rplX_A0A1C7B7C3 | large ribosomal subunits (Ribosomal 50S) |
| rpmE_A0A2R3J325 | large ribosomal subunits (Ribosomal 50S) |
| rpmE2_A0A2R3IY21 | large ribosomal subunits (Ribosomal 50S) |
| rpmE2_A0A2R3IY21 | large ribosomal subunits (Ribosomal 50S) |
| rpmG_A0A0H2ZJ31 | large ribosomal subunits (Ribosomal 50S) |
| ***> 2-fold decrease*** | ***Targets shared with polymyxins*** |
| fabG_A0A2X2AX16 | LPS biosynthesis |
| lpxC_A0A2R3J0I2 | lipid A biosynthesis |
| ***> 2-fold decrease*** | ***Targets shared with quinolones*** |
| pmbA_A0A485GYR1 | modulator of DNA gyrase (PmbA/TldE family) |
| ***> 2-fold decrease*** | ***Antibiotic resistance*** |
| armR_A0A1G7UIG4 | anti-repressor ArmR (aminoglycoside impermeability) |
| blaPDC_A0A173G7Y2 | oxyimino-cephalosporin resistance |
| capD_A0A4P0UDQ9 | multidrug resistance |
| rlmB_A0A431XAU8 | 23S rRNA (guanosine-2'-O-)-methyltransferase (aminoglycoside resistance) |
| yxaF_2_A0A2R3J354 | multidrug resistance transporter; homologous to LmrA |
